# Iron Availability Increases the Pathogenic Potential of *Salmonella Typhimurium* and Other Enteric Pathogens at the Intestinal Epithelial Interface

**DOI:** 10.1371/journal.pone.0029968

**Published:** 2012-01-17

**Authors:** Guus A. M. Kortman, Annemarie Boleij, Dorine W. Swinkels, Harold Tjalsma

**Affiliations:** Department of Laboratory Medicine, Nijmegen Institute for Infection, Inflammation and Immunity (N4i) & Institute for Genetic and Metabolic Disease (IGMD) of the Radboud University Nijmegen Medical Centre, Nijmegen, The Netherlands; Charité-University Medicine Berlin, Germany

## Abstract

Recent trials have questioned the safety of untargeted oral iron supplementation in developing regions. Excess of luminal iron could select for enteric pathogens at the expense of beneficial commensals in the human gut microflora, thereby increasing the incidence of infectious diseases. The objective of the current study was to determine the effect of high iron availability on virulence traits of prevalent enteric pathogens at the host-microbe interface. A panel of enteric bacteria was cultured under iron-limiting conditions and in the presence of increasing concentrations of ferric citrate to assess the effect on bacterial growth, epithelial adhesion, invasion, translocation and epithelial damage *in vitro*. Translocation and epithelial integrity experiments were performed using a transwell system in which Caco-2 cells were allowed to differentiate to a tight epithelial monolayer mimicking the intestinal epithelial barrier. Growth of *Salmonella typhimurium* and other enteric pathogens was increased in response to iron. Adhesion of *S. typhimurium* to epithelial cells markedly increased when these bacteria were pre-incubated with increasing iron concentration (*P* = 0.0001), whereas this was not the case for the non-pathogenic *Lactobacillus plantarum* (*P* = 0.42). Cellular invasion and epithelial translocation of *S. typhimurium* followed the trend of increased adhesion. Epithelial damage was increased upon incubation with *S. typhimurium* or *Citrobacter freundii* that were pre-incubated under iron-rich conditions. In conclusion, our data fit with the consensus that oral iron supplementation is not without risk as iron could, in addition to inducing pathogenic overgrowth, also increase the virulence of prevalent enteric pathogens.

## Introduction

Iron is a highly abundant metal on earth and is vital for virtually all organisms. Despite its abundance, iron deficiency is the most prevalent nutrition disorder worldwide. It mostly affects infants, young children and women in developing countries. Iron deficiency has major health consequences such as infection, poor pregnancy outcome, and impaired physical and cognitive development [1]. Several trials have shown that iron deficiency can be effectively controlled by both iron supplementation and fortification programmes [2]. However, safety of iron supplementation has been questioned and there is evidence suggesting that untargeted oral iron supplementation in regions with high prevalence of malaria transmission and infectious diseases can cause an increase in infections, hospital admission and mortality in young children [3], [4], [5]. This might be at least partly ascribed to iron also being an essential requirement for the growth of most bacterial species. Importantly, iron availability is frequently involved in the expression of virulence-associated properties in pathogenic bacteria [6], [7].

The human gut is the natural habitat for a large and dynamic bacterial community. Major functions of the gut microflora include important trophic effects on intestinal epithelia, on immune structure and function, and protection of the colonized host against invasion by pathogenic microbes [8]. It has been described that dietary ferric iron and iron deprivation can influence the microflora composition of the mouse intestine [9], [10]. Very recently, Zimmermann et al. showed in a study among African children that iron fortification caused a potentially more pathogenic gut microbiota profile (i.e. increased relative abundance of pathogenic species) [11]. This was predominantly the case for *Salmonella* spp., which are capable of invading human epithelial cells, translocate across the colonic wall, and subsequently can cause systemic disease [11], [12], [13]. The increase in infections upon oral iron supplementation that was reported by Sazawal et al. [3] might partly originate from such pathogenic shifts in the colon microflora due to high concentrations of unabsorbed iron during treatment. Abundance of pathogenic enterobacteria after oral iron supplementation might cause diarrhea and a systematic review of Gera and Sachdev indeed reported a slight increase in the risk of developing diarrhea upon oral iron administration [14]. Importantly, diarrhea is most often a sign of gastrointestinal infection and is a major cause of morbidity and mortality among young children worldwide [15]. Therefore, a prior recommendation of both the WHO and the U.S. National Institutes of Health Technical Working Group (NHI TWG) is to investigate the impact of iron preparations on the gut microflora [16].

Roughly, there are two factors - directly driven by luminal iron - which may act together in gut borne infections: i) decrease in epithelial integrity and ii) increase in pathogen growth and virulence. Decrease in epithelial integrity has been reported *in vitro* and *ex vivo* and the corresponding increased permeability of the intestine may provide a portal of entry for opportunistic enteric pathogens [17], [18], [19]. However, little is known about the direct effects of luminal iron on the growth and virulence of enteric pathogens. Therefore, the aim of this study was to investigate how the pathogenic potential of gut bacteria is modulated by iron *in vitro*. To this purpose, bacterial adhesion, invasion and translocation characteristics of a panel of enteric pathogens was investigated using differentiated monolayers of the intestinal epithelial cell lines Caco-2 and E12 as a model for the gut epithelium.

## Materials and Methods

### Bacterial strains, media and growth conditions

The strains used in this study were: *Salmonella typhimurium NTB6, Escherichia coli NTB5, Enterococcus faecalis ATCC 19433, Lactobacillus plantarum WCFS1* [20] and *Citrobacter freundii NTBK1*. These bacteria were cultured at 37°C/5%CO_2_ in Iscove's Modified Dulbecco's Medium (IMDM, Invitrogen). This chemically defined medium does not contain iron in its formulation. For *L. plantarum* the medium was supplemented with 30 mg/L MnCl_2_·4H_2_O, 1 g/L sodium acetate and 10 mmol/L HCl. To determine the effect of iron on bacterial growth, fresh IMDM medium with increasing ferric citrate (Sigma-Aldrich) concentrations (0–1000 µmol/L) was inoculated with a fresh overnight culture. To monitor bacterial growth, the optical density was periodically measured at 620 nm (OD_620_).

### Cell line, media and growth conditions

The colon adenocarcinoma cell line Caco-2 (obtained from the American Type Culture Collection) was cultured under standard conditions in DMEM (Lonza) supplemented with 10% heat-inactivated FBS (Invitrogen), 20 mmol/L HEPES, 100 nmol/L nonessential amino acids (Invitrogen), and 2 mmol/L L-glutamine (Lonza). The cells were subcultured every 6 days and used between passage numbers 2–25. A description of the culturing of mucus producing E12 cells is available from the Online Supporting Materials and methods S1.

### Bacterial adhesion and invasion assay

The influence of iron on adhesion and invasion of the mentioned bacterial strains was studied by the use of the following adhesion assay.

#### Pre-incubation of bacteria with ferric citrate

IMDM with increasing ferric citrate concentrations (0–50 µmol/L) was inoculated with overnight cultures and grown to exponential phase. The bacterial cells were pelleted, resuspended and concentrated in IMDM with 10% glycerol for storage at −80°C until use. Serial dilutions of thawed stocks were transferred to blood agar plates and incubated overnight to determine the colony forming units (CFU).

#### Culturing of Caco-2 cells

Caco-2 cells were subcultured in a 24-well plate and maintained until use in adhesion and invasion assays. The assays were performed on confluent monolayers between 13–21 days after seeding the cells.

#### Bacterial adhesion and invasion assay

The Caco-2 monolayers were washed once with PBS. The stocks of bacterial strains that were grown in IMDM with or without ferric citrate were pelleted and resuspended in IMDM. Next, bacteria were added to the monolayers at a multiplicity of infection (MOI) of 10∶1 in IMDM followed by incubation for 2 h at standard conditions. To determine the number of adherent bacteria, monolayers were washed three times with PBS, cells were trypsinized and lysed with ice-cold PBS containing 0.025% Triton X-100. Serial dilutions of cell lysates were transferred to blood-agar plates for CFU counting. To determine the number of invaded bacteria, monolayers were washed three times with PBS, incubated for another 1.5 h and subsequently incubated with 200 mg/L gentamycin (Invitrogen)+50 mg/L ampicillin (Calbiochem) for 1 h at standard conditions to kill extracellular bacteria. Subsequently, the monolayers were washed twice with PBS, trypsinized and lysed for CFU counting as described above [21].

### Bacterial translocation assay

Caco-2 cells were allowed to grow and differentiate in 21 days to a polarized tight monolayer on the membrane of a Transwell® Permeable Support (12 wells, 12 mm insert) with 3.0 µm polycarbonate membrane (Corning) under standard culture conditions. At day 21, the wells and inserts were washed once with PBS and IMDM was added. To check for the monolayer integrity, the trans epithelial electrical resistance (TEER) was measured with the use of the Millicell®-ERS (Millipore). Bacteria that were or were not pre-loaded with iron (as described above) were apically added to the monolayers at a MOI of 10∶1 in IMDM. Infected cells were incubated at standard conditions and monolayer integrity was monitored by periodical TEER measurements. After 2 h both compartments were washed three times with PBS and fresh IMDM was added. The incubation of infected cells was continued for 2 h after which a sample of the lower compartment was taken to determine the amount of translocated bacteria by CFU counting.

### Determination of LDH-release of epithelial cells upon bacterial infection

To investigate the detrimental effect of enteric bacteria to Caco-2 monolayers, the lactate dehydrogenase (LDH) release into the growth medium was determined. Media from adhesion experiments were collected after 2 h of incubation. Samples were spun for 15 min at 16,100× g at 4°C. Supernatants were used in the Cytotox 96® Non-Radioactive Cytotoxicity Assay (Promega) according to the manufacturer's protocol.

### Statistical analysis

Each adhesion/invasion and translocation experiment was performed with 2–3 biological replicates, repeated up to 3 times on separate days and results were expressed as mean+SD. To compare the means, one-way ANOVA with Tukey's post-hoc test (for comparison of >2 means with equal variances, as assessed by Bartlett's test or F-test) or an unpaired t-test (2-tailed) (for comparison of 2 means) was used. In case of unequal variances (as assessed by F-test), unpaired t-test with Welch's correction was carried out. Analysis was performed using GraphPad Prism version 4.00 for Windows, GraphPad Software, San Diego California USA. *P*-values<0.05 were considered statistically significant and *P*-values<0.10 were considered as an important significance level.

## Results

### *In vitro* growth of enteric bacteria

**Figure 1:**
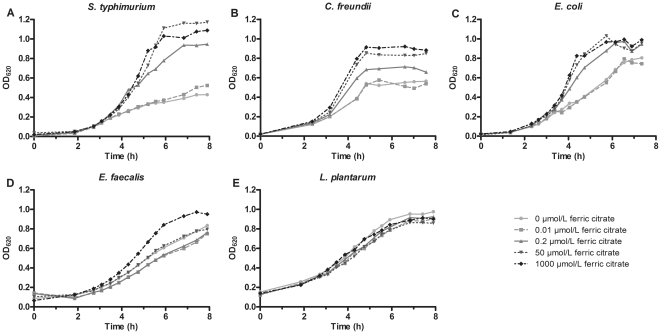
Effect of iron on growth of enteric bacteria. Effect of various concentrations of ferric citrate on *in vitro* growth of (**A**) *S. typhimurium*, (**B**) *C. freundii*, (**C**) *E. coli*, (**D**) *E. faecalis* and (**E**) *L. plantarum*.

To investigate whether pathogenic bacteria have a potential growth advantage over non-pathogenic bacteria in an iron-rich environment, the effect of ferric citrate on growth of a selection of enteric bacteria was tested *in vitro* (**Figure 1**). These experiments revealed a clear concentration-dependent growth stimulatory effect in iron-supplemented growth medium for the pathogen *S. typhimurium* and the opportunistic pathogens *C. freundii* and *E. coli* (**Figure 1A–C**). Only a small beneficial effect was noted for the opportunistic pathogen *E. faecalis*, while iron did not influence growth of the non-pathogenic commensal *L. plantarum* (**Figure 1D and E**).

### Adhesion of enteric bacteria to an epithelial monolayer

**Figure 2:**
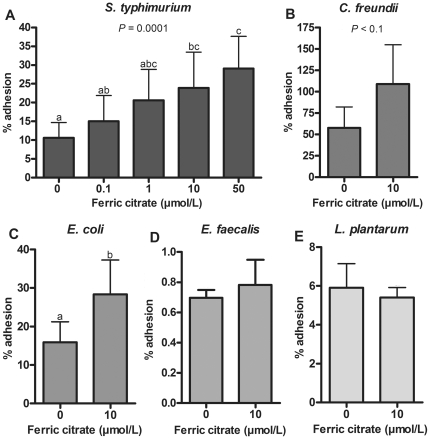
Effect of iron on bacterial adhesion to an epithelial monolayer. Adhesion (mean+SD) of enteric bacteria to a monolayer of Caco-2 cells is given as percentage of the inoculum. **A**: *S. typhimurium*, *n* = 8. **B**: *C. freundii*, *n* = 4. **C**: *E. coli*, *n* = 6. **D**: *E. faecalis*, *n* = 6. **E**: *L. plantarum*, *n* = 5. Means without a common letter differ, *P*<0.05. Notably, adhesion data of *S. typhimurium* were derived from 4 separate experiments performed at 13, 15, 18 and 21 days post-seeding of Caco-2 cells. The fact that each experiment revealed the same trend is indicative for similar physiochemical properties of the monolayer at these time points.

Adhesion to host epithelial cells is an important virulence characteristic for pathogenic bacteria. To test the contribution of iron availability to bacterial adhesion, bacteria were pre-incubated with ferric citrate and added to epithelial monolayers under iron-limiting conditions, after which the percentage adhesion of the inoculum was determined. As shown in **Figure 2A**, adhesion of *S. typhimurium* significantly increased with increasing iron concentration (one-way ANOVA: *P* = 0.0001, 0 µmol/L vs. 10 µmol/L: *P*<0.05). This increase was not due to differential growth since the CFU did not differ among the 1–50 µmol/L ferric citrate conditions during the time course of this experiment. The opportunistic pathogen *C. freundii* tended to adhere more (*P* = 0.097) and *E. coli* adhered more (*P* = 0.014) after pre-incubation with 10 µmol/L ferric citrate compared to bacteria pre-incubated under iron-limiting conditions (**Figure 2B and C**). Adhesion of the opportunistic pathogen *E. faecalis* was not influenced by pre-incubation with iron (**Figure 2D**). Interestingly, the non pathogenic commensal *L. plantarum* (**Figure 2E**) displayed a slight, but non-significant decrease in adhesion characteristics in response to an increase in iron availability.

Mucus, covering the human intestinal epithelium, could influence bacterial adhesion. Therefore, we investigated whether iron availability could also increase the adhesion of *S. typhimurium* to mucus producing E12 cells. The latter cell line is derived from HT29-MTX and capable of forming tight monolayers covered with an adherent mucus layer [22]. For all conditions the adhesion of *S. typhimurium* to E12 cells was much higher compared to Caco-2 cells (data not shown). Importantly, also in this case the tendency towards increased adhesion of bacteria that were pre-incubated with increased iron concentrations was found to be statistically significant (*P*<0.05; **Figure S1A**). The latter observation indicates that a protective mucus layer does not prevent the increased adhesion of iron-loaded bacteria to intestinal epithelial cells.

### Invasion of enteric bacteria into epithelial cells

**Figure 3:**
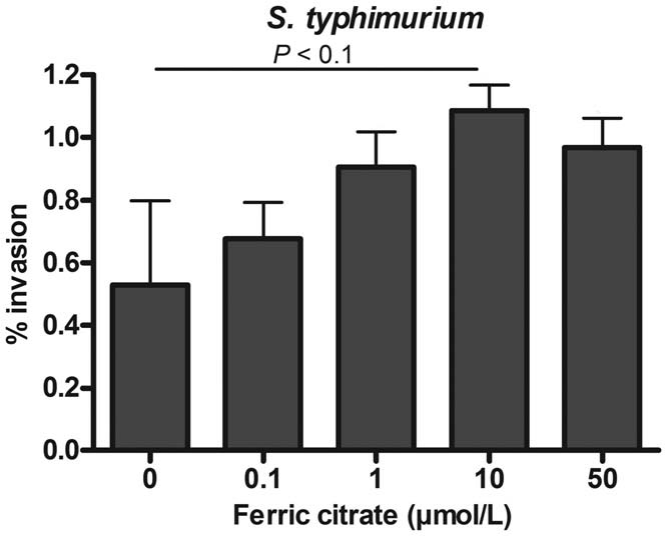
Effect of iron on invasion of *S. typhimurium* into epithelial cells. Invasion (mean+SD) of *S. typhimurium* into Caco-2 cells, *n* = 2. Invasion after 3.5 h is given as percentage of the inoculum. The inoculum was removed after 2 hours of adhesion time. Means of 0–10 µmol/L ferric citrate were compared by one-way ANOVA.

During the infectious process, bacterial adhesion can be followed by invasion into intestinal epithelial cells. Therefore, the effect of pre-incubation with ferric citrate on cell invasion was assessed for *S. typhimurium*, *E. faecalis* and *L. plantarum*. Of these strains, only *S. typhimurium* was able to substantially invade a differentiated monolayer of epithelial cells. Similar to adhesion, invasion of *S. typhimurium* tended to increase with increasing iron concentration up to 10 µmol/L ferric citrate (One-way ANOVA, *P* = 0.09; **Figure 3**). However, this trend disappeared when the percentage bacterial invasion was calculated as a function of the adherent bacteria (**Figure S2**). This indicates that the increase in invasion is merely a consequence of increased adhesion of *S. typhimurium*, and that invasion itself is not largely influenced by iron availability. Notably, the invasion of bacteria pre-incubated with 50 µmol/L ferric citrate was unexpectedly low in relation to the increased adhesion under these conditions.

### Translocation of enteric bacteria across an epithelial monolayer

**Figure 4:**
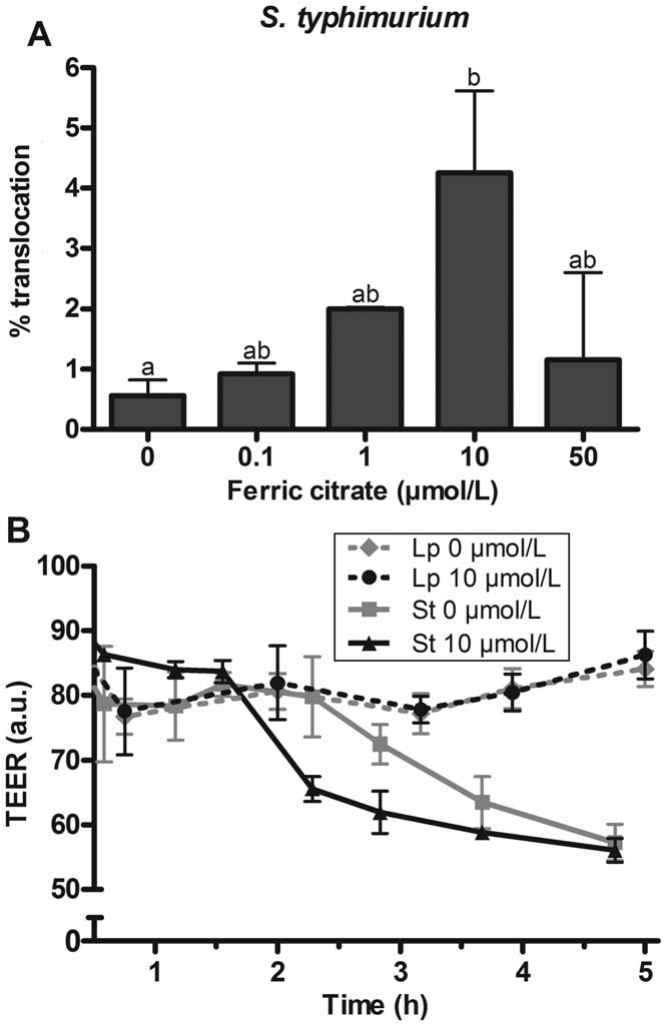
Effect of iron on the ability of *S. typhimurium* to cross and deteriorate an epithelial monolayer. Effect of iron on the ability of *S. typhimurium* to cross an epithelial monolayer of Caco-2 cells and the integrity of this monolayer. **A**: The translocation is given as percentage of the inoculum (mean+SD), *n* = 2. Means without a common letter differ, *P*<0.07. **B**: The integrity of the Caco-2 monolayer during *S. typhimurium* (St) and *L. plantarum* (Lp) translocation, monitored by TEER measurements.

To cause a systemic infection, enteric bacteria first need to breach the intestinal epithelial barrier. Therefore, the ability of *S. typhimurium* to translocate across an epithelial monolayer was investigated as a function of iron availability. As shown in **Figure 4A**, translocation of *S. typhimurium* clearly increased with increasing iron availability up to 10 µmol/L ferric citrate (10 µmol/L vs. iron deplete control *P* = 0.06). Interestingly, translocation sharply decreased when bacteria were pre-incubated with 50 µmol/L ferric citrate (**Figure 4A**). As a control experiment, the translocation of *L. plantarum* was assessed. Translocation of this bacterium was neglectably low and not stimulated by increased iron availability (data not shown).

The ability of *S. typhimurium* to translocate across a mucus covered epithelial layer of E12 cells was also investigated. These experiments showed that also in this case, the translocation of *S. typhimurium* across E12 cells increased (*P*<0.05) when the bacteria were pre-incubated with increasing concentrations of ferric citrate (**Figure S1B**). These data indicate that a protective mucus layer does not abolish the iron-dependent increase in translocation efficiency of *S. typhimurium* across an epithelial monolayer (**Figure 4A**).

### Effect of iron-loaded bacteria on epithelial integrity and cell cytotoxicity

**Figure 5:**
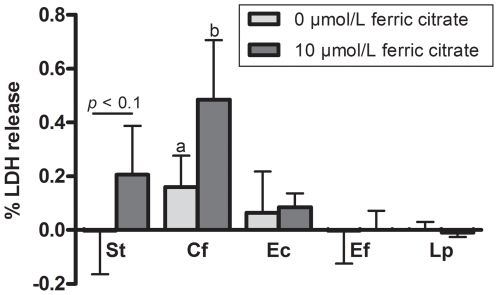
Effect of iron on the ability of enteric bacteria to induce cell damage. LDH-release (mean+SD) as a measure of cell damage of Caco-2 cells upon co-incubation with *S. typhimurium* (St, *n* = 5), *C. freundii* (Cf, *n* = 4), *E. coli* (Ec, *n* = 4), *E. faecalis* (Ef, *n* = 4), and *L. plantarum* (Lp, *n* = 2) pre-incubated with or without ferric citrate. The percentage LDH release compared to the control (no bacteria) was corrected for the number of bacteria in the medium (average between t = 0 and t = 2 h). Means within a group and without a common letter differ significantly, *P*<0.05.

To monitor epithelial integrity during the translocation experiments, the electrical resistance of the monolayer was monitored by TEER measurements at regular intervals. These experiments showed that incubation with *S. typhimurium* resulted in decreased TEER values in time (**Figure 4B**). As expected, the deterioration rate of the epithelial integrity increased when *S. typhimurium* was pre-incubated with iron as depicted for the 10 µmol/L ferric citrate condition. In contrast, the integrity of the monolayer remained unaffected upon incubation with other tested bacteria, irrespective of a pre-incubation step with ferric citrate, as illustrated for *L. plantarum* (**Figure 4B**). As a second indicator of epithelial damage, the cellular LDH release was determined after enteric bacteria were allowed to adhere to the monolayer for 2 hours. As shown in **Figure 5**, epithelial cells tended to release more LDH in response to *S. typhimurium* that was pre-incubated with 10 µmol/L ferric citrate compared to the 0 µmol/L condition (*P* = 0.09). This is in line with the increased deterioration rate under these conditions as monitored by TEER measurements. Interestingly, the LDH release in response to *C. freundii* pre-incubated with 10 µmol/L ferric citrate was also greater compared to *C. freundii* grown in iron deplete medium (*P* = 0.018), despite the fact that the TEER did not drop under these conditions (data not shown). The latter observation implies that LDH release is an early marker for epithelial damage, which may correlate with adhesion of pathogenic bacteria (see **Figure 2B**). In this respect, the *E. coli* strain used in this study seems not very hostile since the TEER did not drop and LDH release did not increase despite an increased adhesion after pre-incubation with 10 µmol/L ferric citrate. Contrarily, the LDH release in response to *E. faecalis* and *L. plantarum* was low compared to the release in response to the other bacteria, and unaffected by pre-incubation of these bacteria with ferric citrate. The latter observation is consistent with the fact that adhesion of these bacteria remained unaffected under iron rich conditions (**Figure 2D and E**).

## Discussion

The safety of iron supplementation and fortification programmes in developing countries has been questioned [4]. Very recently it has been shown that biscuits fortified with low bioavailable electrolytic iron caused an increase in fecal enterobacteria (predominately *Salmonella* spp.) in African children [11]. This shift towards a more unfavorable number of enteric pathogens might be one of the origins of the increase in infections, hospital admissions and mortality which was found by Sazawal et al. [3]. Here we investigated the relative effects of iron on growth and virulence traits of enteric pathogens and commensals.

Our *in vitro* experiments showed that the switch from iron-limiting to iron rich conditions resulted in an enhanced growth of *S. typhimurium* and other enteric pathogens. On the other hand, growth of *L. plantarum* that is not strictly dependent on iron [23], was not enhanced by iron. These results fit with the finding that enteric pathogens have the potential to outgrow the commensal population when large amounts of unabsorbed dietary iron enter the colon *in vivo* [11].

The ability to replicate is important for all bacteria, but to establish an infection, pathogens first need to adhere to the colonic wall. Our current study clearly showed that iron availability increased adhesion of enteric pathogens to intestinal epithelial cells *in vitro*, which was most prominently observed for *S. typhimurium*. Importantly, the increased adhesion of *S. typhimurium* as a function of iron was not only observed with Caco-2 cells, but also in case of mucus producing intestinal epithelial E12 cells that may more closely resemble the *in vivo* situation. In contrast, iron did not affect adhesion of *E. faecalis* and even seemed to slightly reduce the adhesion of *L. plantarum* to epithelial cells. Importantly, these results indicate that in addition to a growth advantage of enteric pathogens, iron also has the potential to contribute to increased colonization of these enteric pathogens to the colonic wall. *In vivo*, colonization of enteric pathogens depends on many factors, among which the colonization resistance of the resident commensal population. In this respect, Stecher et al. postulated the interesting “*like will to like*” concept, based on the observation that mice with relatively high *E. coli* densities in their intrinsic intestinal population were more susceptible to *Salmonella* infections [24]. It may therefore be envisaged that iron can also indirectly enhance *S. typhimurium* infections *in vivo* by increasing intestinal colonization with related commensals.

Translocation across the colonic wall is the third step in establishing a gut-borne infection. Our current *in vitro* data show that iron availability increases the cell invasion and epithelial translocation potential of *S. typhimurium*. However, the increase in invasion seems to be a direct consequence of the increased adhesion, which implies that there is no stimulatory effect of iron on invasion itself. This could indicate that the type III secretion system (T3SS) of *S. typhimurium*, which is directly involved in the invasion of epithelial cells, was not further induced by ferric citrate under the applied experimental conditions. Nevertheless, other studies have shown that T3SS is induced by iron through the ferric uptake regulator (Fur) [25], [26], [27]. Interestingly, invasion even appeared slightly less efficient when *S. typhimurium* was pre-incubated in 50 µmol/L ferric citrate, suggesting that certain invasion-specific factors become affected under excessive iron conditions. *S. typhimurium* initially invades directly into host cells and can hereby affect tight junction complex proteins [13], [28], [29]. This affects epithelial integrity and subsequently provides the opportunity for *S. typhimurium* to cross the epithelium via the paracellular route [29]. The reduced translocation efficacy at excessive iron conditions could therefore fit with impaired cell invasion of *S. typhimurium* during the initial phases of infection. Besides enteroinvasive strains like *S. typhimurium*, there are many other enteric pathogens that do not translocate across the bowel wall, but can cause severe intestinal inflammation. The virulence of such pathogens is also likely to be positively influenced by increased iron availability.

Our experiments showed that pre-incubation of the enterobacteria *S. typhimurium* and *C. freundii* with iron increased damage to epithelial cells as measured by LDH release. This may very well be associated with the increased adhesion of these bacteria under these conditions. However, the release of bacterial products such as toxins, which was not assessed in this study, could differ between high and low iron conditions and can also play a role in the increased epithelial cell damage. Furthermore, iron-loading of *S. typhimurium* resulted in faster deterioration the epithelial integrity of a monolayer (TEER measurement), which was associated with increased bacterial translocation up to 10 µmol/L ferric citrate. Since high TEER values mainly represent the existence of tight junctions [29], this is in line with a hypothetical model in which *S. typhimurium* actively affects tight-junction complexes, which is stimulated upon increased invasion of epithelial cells under iron-rich conditions. We want to emphasize that all adhesion, invasion and translocation assays were performed with bacteria that were pre-loaded with iron, but that the experiments themselves were performed under iron-limiting conditions. This was important to prevent bias in our data, as it has been reported that iron itself has the potential to damage the gut wall directly via generation of oxygen radicals [17], [30]. In our current study, Caco-2 cells and E12 cells were maintained under standard condition without any additional iron, which is different from the study design of Foster et al. who showed that elevated iron status of enterocytes increased bacterial invasion [31]. Clearly, our study adds that enteric pathogens themselves have the potential to benefit from increased luminal iron availability, already during the initial phases of infection (i.e. cell adhesion and cell damage).

In summary, our *in vitro* data support the hypothesis that luminal iron from oral iron supplementation or fortification can increase growth and virulence of enteric pathogens. It goes without saying that an animal infection model is required for future validation of our data in a more complex situation. Nevertheless, our study clearly supports the current idea that nutritionist should be aware of the potential harmful effects of oral iron supplementation in areas with high infection pressure, as present in developing countries. The ideal safe iron preparation should be low dose and highly bioavailable for humans, while it is difficult to access for enteric pathogens. Research to the latter part is currently underexposed, but certainly deserves more attention in the light of the recent awareness of the risk of general iron supplementation programmes. In addition, it is important to develop point-of-care diagnostic tools to discriminate between individuals with iron deficiency anemia (IDA) who will directly benefit from iron supplementation and those with anemia due to chronic infection (ACD). In the latter case, anemia is (also) a result from the human iron-withdrawal strategy mediated by increased circulating levels of the iron-regulatory hormone hepcidin upon (malarial) infection [32], [33]. Subsequently, hepcidin not only blocks iron release from the reticulo-endothelial macrophages but it also inhibits absorption from the intestine [32], [34], meaning that luminal pathogens will benefit more from dietary iron supplementation than the host itself. Reliable on-site discrimination between ACD and IDA may contribute to the safe supplementation of iron. From a mechanistic point of view, several questions remain to be answered, such as the identification of the enterobacterial factors that are responsible for the increased adhesion under iron-rich conditions. This and other questions will be subject of our ongoing investigations.

## Supporting Information

Figure S1 **Effect of iron on the ability of S. typhimurium to adhere to, and translocate across, an epithelial monolayer of E12 cells covered with mucus.** In vitro adhesion (mean+SD) to E12 cells, and translocation (mean+SD) of S. typhimurium across a monolayer of E12 cells. A: Because adhesion to E12 cells was much higher than adhesion to Caco-2 cells, the number of adherent bacteria to E12 cells was expressed as percentage of the average CFU (CFU at start – CFU after 2 h) in the culture medium, n = 2. B: Translocation is given as percentage of the average CFU (CFU at start – CFU after 2.5 h) in the culture medium, n = 3. Means without a common letter differ P<0.05.
(TIF)

Figure S2 **Effect of iron on invasion of S. typhimurium into Caco-2 epithelial cells.** Invasion (mean+SD) of S. typhimurium into Caco-2 epithelial cells. Invasion after 3.5 hours is given as percentage invaded bacteria of the adherent bacteria at the 2 hour time point, n = 2.
(TIF)

Materials and Methods S1 Supporting materials and methods.
(DOC)
