# Supplementary figures and images for: Iron Availability Increases the Pathogenic Potential of Salmonella Typhimurium and Other Enteric Pathogens at the Intestinal Epithelial Interface
Source: PLoS One. 2012 Jan 17;7(1):e29968. doi: 10.1371/journal.pone.0029968 (PMC3260200; doi:10.1371/journal.pone.0029968)

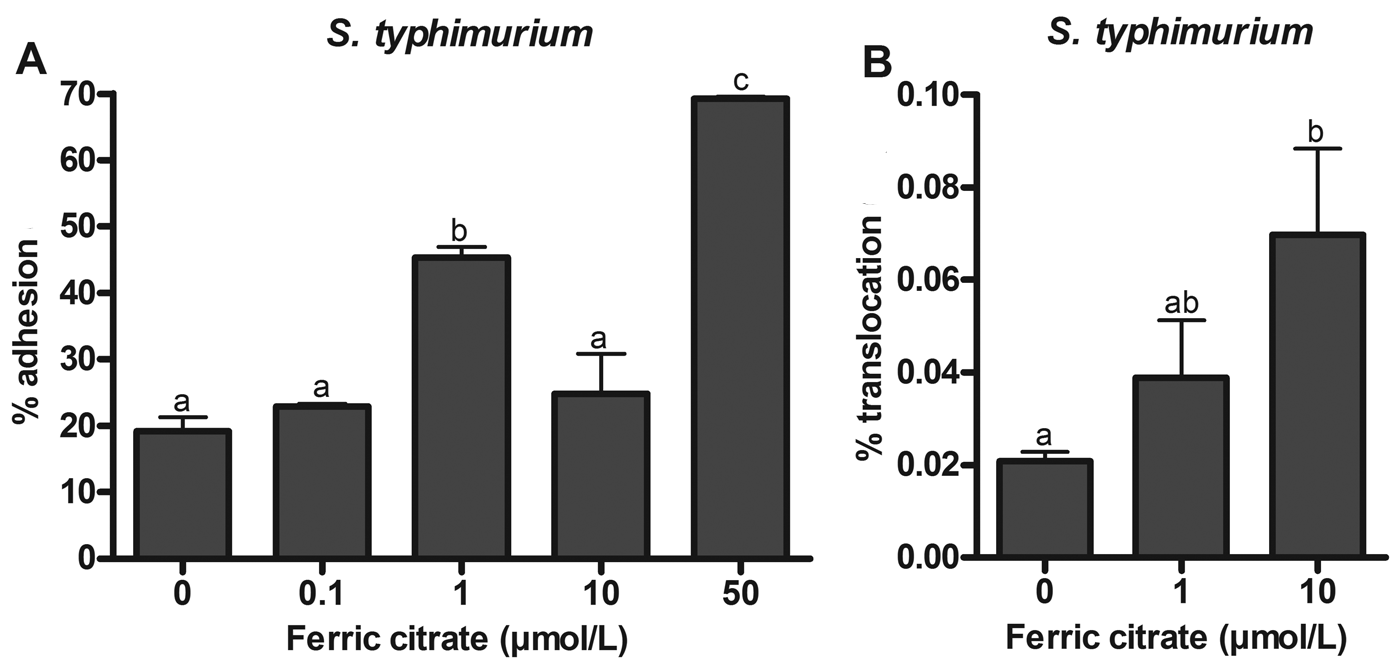

Supplement: Figure S1 — Effect of iron on the ability of S. typhimurium to adhere to, and translocate across, an epithelial monolayer of E12 cells covered with mucus. In vitro adhesion (mean+SD) to E12 cells, and translocation (mean+SD) of S. typhimurium across a monolayer of E12 cells. A: Because adhesion to E12 cells was much higher than adhesion to Caco-2 cells, the number of adherent bacteria to E12 cells was expressed as percentage of the average CFU (CFU at start – CFU after 2 h) in the culture medium, n = 2. B: Translocation is given as percentage of the average CFU (CFU at start – CFU after 2.5 h) in the culture medium, n = 3. Means without a common letter differ P<0.05. (TIF) [file pone.0029968.s001.tif]

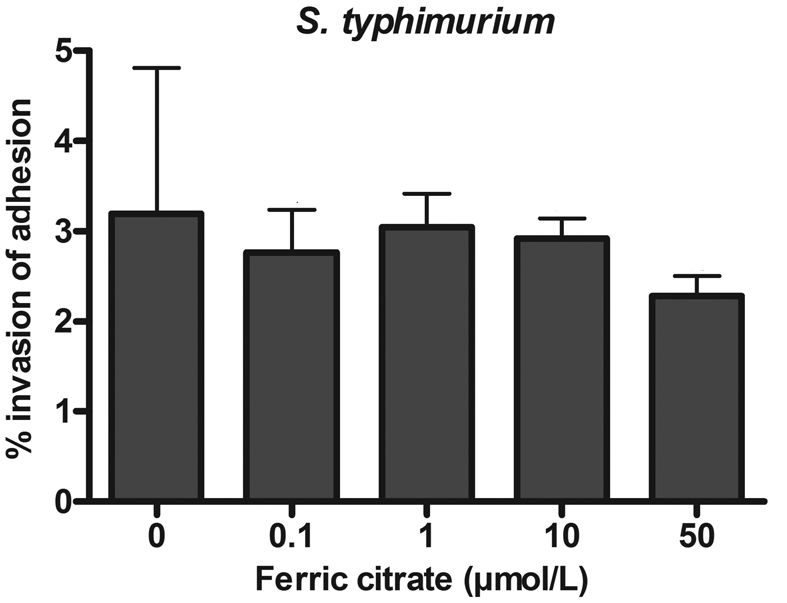

Supplement: Figure S2 — Effect of iron on invasion of S. typhimurium into Caco-2 epithelial cells. Invasion (mean+SD) of S. typhimurium into Caco-2 epithelial cells. Invasion after 3.5 hours is given as percentage invaded bacteria of the adherent bacteria at the 2 hour time point, n = 2. (TIF) [file pone.0029968.s002.tif]
