# Supplementary material for: Iron Availability Increases the Pathogenic Potential of Salmonella Typhimurium and Other Enteric Pathogens at the Intestinal Epithelial Interface
Source: PLoS One. 2012 Jan 17;7(1):e29968. doi: 10.1371/journal.pone.0029968 (PMC3260200; doi:10.1371/journal.pone.0029968)
Supplement: Materials and Methods S1 — Supporting materials and methods. (DOC) [file pone.0029968.s003.doc]

**Iron Availability Increases the Pathogenic Potential of *Salmonella Typhimurium* and Other Enteric Pathogens at the Intestinal Epithelial Interface**

Guus AM Kortman, Annemarie Boleij, Dorine W Swinkels, Harold Tjalsma

**Supplementary information**

**Materials and methods**

Mucus producing cell line E12, media and growth conditions

The human colon carcinoma cell line E12 (1), kindly provided by Dr. Sam Maher, UCD, Dublin, Ireland) was cultured under standard conditions in DMEM (Lonza) supplemented with 10% fetal calf serum (Invitrogen), 20 mmol/L HEPES, 100 nmol/L nonessential amino acids (Invitrogen), 2 mmol/L L-glutamine (Lonza) and Penicillin/Streptomycin (PenStrep, 100 U/ml and 100 µg/ml, respectively) (Invitrogen). The cells were subcultured every 5 days and used between passage numbers 4-6.

Bacterial adhesion assay

The influence of iron on adhesion and translocation of *S. typhimurium* to mucus producing E12 cells was studied by the use of the following adhesion and translocation assay.

**Pre-incubation of bacteria with ferric citrate:** IMDM with increasing ferric citrate concentrations (0-50 µmol/L) was inoculated with overnight cultures and grown to exponential phase. The bacterial cells were pelleted, resuspended and concentrated in IMDM with 10% glycerol for storage at -80˚C until use. Serial dilutions of thawed stocks were transferred to blood agar plates and incubated overnight to determine the colony forming units (CFU).

**Culturing of E12 cells:** E12 cells were allowed to grow and differentiate in 19-20 days to a polarized tight monolayer with an adherent mucus layer on the membrane of a Transwell® Permeable Support (12 wells, 12 mm insert) with 3.0 µm polycarbonate membrane (Corning) under standard culture conditions. 4-7 days before use, culture medium without PenStrep was used. At day 19-20, the wells and inserts were washed once with PBS and IMDM was added.

**Bacterial adhesion and translocation assay:** *S. typhimurium* that was or was not pre-loaded with iron (as described above) was apically added to the monolayers at a MOI of 10:1 in IMDM. Infected cells were incubated at standard conditions. After 2 h a sample of the lower compartments was taken to determine the amount of translocated bacteria by CFU counting. To determine the number of adherent bacteria, monolayers were washed three times with PBS, cells were trypsinized and lysed with ice-cold PBS containing 0.025% Triton X-100. Serial dilutions of cell lysates were transferred to blood-agar plates for CFU counting.

**References**

1. Behrens I, Stenberg P, Artursson P, Kissel T. Transport of lipophilic drug molecules in a new mucus-secreting cell culture model based on HT29-MTX cells. Pharm Res. 2001 Aug;18:1138-45.
